# Acute renal infarction and cardioembolic stroke in a patient with atrial fibrillation and hyperthyroid-induced cardiomyopathy: a case report

**DOI:** 10.1186/s13256-016-0903-x

**Published:** 2016-05-06

**Authors:** Samaj Adhikari, Kumar Paudel, Kamal Pandit, Shovit Thapa, Bikram Prasad Gajurel, Khagendra Dahal

**Affiliations:** 1 Institute of Medicine, Maharajgunj Medical Campus, Tribhuvan University, Kathmandu, Nepal; 2 Department of Medicine, Lakes Region General Healthcare, 80 Highland Street, Laconia, NH USA

**Keywords:** Renal infarction, Cardioembolic stroke, Atrial fibrillation, Hyperthyroidism

## Abstract

**Background:**

Acute renal infarction is a rare entity with varied misleading manifestations resulting in diagnostic delay, misdiagnosis, and treatment leading to renal damage.

**Case presentation:**

We report the case of a 28-year-old Dalit Nepalese man who presented with sudden onset occipital headache and later developed severe left flank pain. He was diagnosed with posterior cerebral infarction with hemorrhagic transformation and a subsequent acute renal infarction with atrial fibrillation and hyperthyroid-induced cardiomyopathy. He was managed with oral anticoagulant and antithyroid drug.

**Conclusion:**

A high index of suspicion of acute renal infarction is required in patients with risk factors of thrombosis presenting sudden onset flank pain.

## Background

Acute renal infarction (ARI) is a rare entity often presenting with misleading manifestations, which may result in its diagnostic delay, misdiagnosis and treatment leading to renal loss [1, 2]. Flank pain, fever, and nausea/vomiting are the commonest presentations of ARI [3]. The causes of ARI are of cardiac origin, secondary to renal artery injury, hypercoagulable disorders, or idiopathic [2]. Atrial fibrillation (AF) is the commonest cardiac arrhythmia that increases the risk of thromboembolism, thereby subsequently leading to cerebral infarctions and the infarctions of other major visceral organs. Patients with cardioembolic stroke have higher likelihood of developing infarctions of visceral organs below the diaphragm with renal infarction being more common [4, 5].

Hyperthyroidism is a common disorder caused by Graves' disease, toxic multinodular goiter, or Hashimoto’s thyroiditis throughout the world, whereas in the developing countries toxic multinodular goiter is a predominant cause of hyperthyroidism along with Graves' disease due to iodine deficiency [6]. Cardiac complications of hyperthyroidism are AF (8 %) and congestive heart failure (6 %) or less commonly dilated cardiomyopathy [7, 8].

We report a rare and interesting case of posterior cerebral infarction with hemorrhagic transformation and a subsequent ARI in a patient with AF and cardiomyopathy due to hyperthyroidism.

## Case presentation

A 28-year-old Dalit Nepalese man came to our emergency department (ED) with sudden onset throbbing occipital headache, which was severe in intensity, continuous and associated with difficulty in vision followed by right-sided retro-orbital pain, three episodes of non-projectile vomiting, and dizziness of 3 days’ duration. He denied loss of consciousness, abnormal body movements, trauma, limbs weakness, altered sensorium, chest pain, palpitation, fever, and cough. No history of diabetes, hypertension, or hyperlipidemia was noted. He was a current smoker of tobacco and an occasional alcohol consumer. No significant family history was noted.

**Fig. 1:**
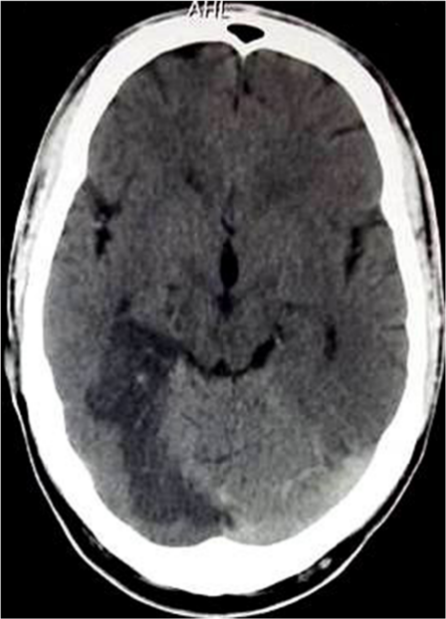
An axial non-contrast head computed tomography showing hypodense area on right occipital lobe consistent with recent posterior cerebral artery infarct

In our ED, his blood pressure was 150/100 mmHg; he had an irregularly irregular pulse rate of 110 per minute, respiratory rate 18 breaths per minute, and body temperature of 38.0 °C. He did not have pallor, icterus, lymphadenopathy, cyanosis, edema, or features of dehydration. His mental state examination did not reveal any deficits. On eye examination, there was bilateral proptosis without impairment of extraocular muscles. His pupils were equally rounded, regular, and reactive bilaterally. Direct and consensual light reflexes were intact. He had right homonymous hemianopia on visual field examination but his color vision was intact. An axial non-contrast head computed tomography (CT) revealed a hypodense area on his right occipital lobe consistent with recent posterior cerebral artery (PCA) infarct (Fig. 1). He was managed with aspirin, atorvastatin, and pain medications.

**Fig. 2:**
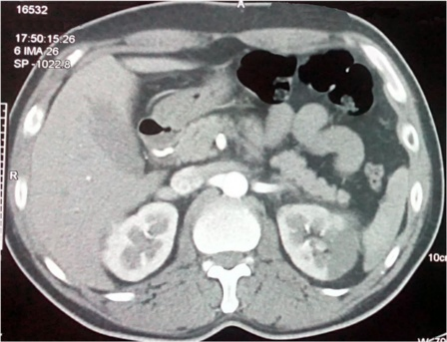
Axial contrast-enhanced computed tomography of abdomen and pelvis showing a non-enhancing, hypodense, sharply demarcated, wedge-shaped area involving the cortex of the patient’s left kidney suggestive of left renal infarction

Two days later, he developed increased severity of headache and sudden onset left flank pain which was severe, non-radiating associated with nausea but without urgency, dysuria, hematuria, fever, and decreased urine output with no history of trauma. A laboratory examination showed normal lactate dehydrogenase (LDH) and white blood cell (WBC) count. His blood urea nitrogen was 4 μmol/L (normal 1.6 to 7 μmol/L ), creatinine 85.0 μmol/L (normal 60 to 130 μmol/L), serum sodium 130 mEq/L (normal 135 to 146 mEq/L), and potassium 3.5 mEq/L (normal 3.5 to 4.2 mEq/L); urine analysis showed WBC of 1 to 2 per high-power field (HPF), no red blood cells (RBCs) per HPF, epithelial cells of 4 to 6 per HPF, and proteinuria (albumin 1+) without any casts and crystals. His platelet count was 212,000/mm^3^. His bleeding time was 2.15 minutes (normal 1 to 5 minutes), clotting time 6.30 minutes (normal 4 to 9 minutes), prothrombin time 15 seconds (control 14 seconds) with international normalized ratio (INR) of 1.07, and activated partial thromboplastin time was 30 seconds (control 30 seconds). A thyroid function test showed: free triiodothyronine 13.7 pmol/L (normal 4.26 to 8.1 pmol/L), free thyroxine 70.4 pmol/L (normal 10.2 to 28.2 pmol/L), and thyroid-stimulating hormone <0.015 μIU/ml (normal 0.46 to 4.68 μIU/ml). A liver function test revealed serum bilirubin 12.6 μmol/L (normal 3 to 21 μmol/L), direct bilirubin 2.3 μmol/L (normal 0 to 6 μmol/L), serum glutamic pyruvic transaminase (SGPT) 28 U/L (normal 5 to 40 U/L), serum glutamic oxaloacetic transaminase (SGOT) 51 U/L (normal 5 to 40 U/L), and alkaline phosphatase (ALP) 179 U/L (normal 64 to 306 U/L); his serology was negative for HIV, hepatitis B surface antigen (HBs Ag), and anti-hepatitis C virus (HCV) antibody. A 12-lead electrocardiogram (ECG) showed AF and two-dimensional cardiac echocardiography showed dilated cardiac chambers, moderate left ventricular ejection fraction (LVEF = 35 to 40 %), and mild mitral and tricuspid regurgitation without clots, vegetations, or pericardial effusion.

**Fig. 3:**
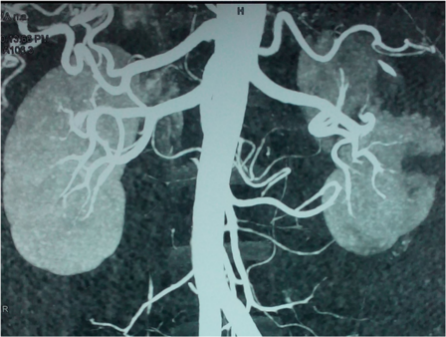
Computed tomography angiogram of abdomen revealing multiple wedge-shaped infarcts in left renal parenchyma with occlusion in interlobar branch of left renal artery supplying the mid-pole

**Fig. 4:**
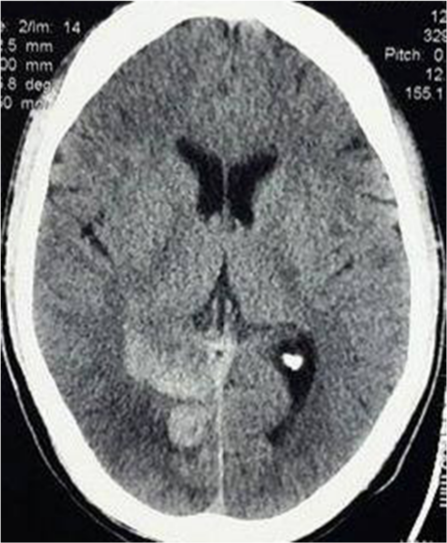
Axial non-contrast computed tomography of head showing grade II hemorrhagic transformation on the left occipital lobe

Axial contrast-enhanced CT images of his abdomen and pelvis showed a non-enhancing, hypodense, sharply demarcated, wedge-shaped area involving the cortex of his left kidney suggestive of left renal infarction (Fig. 2); this was followed by a CT angiogram of his abdomen revealing multiple wedge-shaped infarcts in left renal parenchyma with occlusion in the interlobar branch of his left renal artery supplying the mid-pole (Fig. 3). As more than half of his PCA territory was involved in a CT scan of his brain and he had new onset severe headache, a repeat axial non-contrast CT of his head was performed which showed grade II hemorrhagic transformation on his left occipital lobe (Fig. 4) precluding the use of urgent anticoagulation. Thrombectomy, endovascular thrombolysis, and surgical thrombolysis were not performed due to the situation of his thrombus and involvement of a relatively small-sized deep artery. He was managed conservatively. Once resolution of hemorrhagic transformation was confirmed with a repeat CT scan, he was started on warfarin for anticoagulation; he was carefully monitored for evidence of bleeding. His hyperthyroidism was managed with carbimazole and a beta blocker. Upon discharge, his symptoms were resolved and acute kidney injury had normalized.

## Discussion

ARI is a rare condition with prevalence of 7 per 100,000 among the patients visiting ED while in an autopsy series it was found in 10.2 % of patients with fatal stroke [4, 9]. Owing to the nonspecific symptoms of ARI, it is often misdiagnosed as other conditions namely renal colic, pyelonephritis, renal carcinoma, or infective endocarditis [3].

Patients with suspected ARI need hematological investigations and urine analysis positive for RBCs. The most consistent abnormalities reported in the literature are elevated serum LDH, hematuria, and leukocytosis [3, 10], which are often nonspecific and could be present in mesenteric ischemia/infarction, myocardial infarction (MI), urinary tract infection (UTI), or hemolysis. Despite the nonspecific nature of these findings, a high index of suspicion is needed in patients with these derangements who have AF. Rheumatic heart disease with mitral stenosis, hypertension, hyperthyroidism, obesity, chronic kidney disease, dilated cardiomyopathy, or other cardiopulmonary conditions may predispose patients to AF that may result in thromboembolism leading to cerebral, renal, mesenteric, or splenic infarctions. Patients who have a cerebrovascular event secondary to cardiac emboli due to AF ought to be closely monitored for any clinical signs of visceral infarction thereafter.

Hazanov *et al*. studied 44 cases of renal infarction secondary to AF and found 54 % of patients to have hematuria and 93 % elevated LDH [10]. Frank hematuria or elevated serum LDH were absent in this patient; however, proteinuria was detected in a urine analysis of our patient as in other case reports in the literature [11, 12]. The imaging studies that can be used to diagnose renal infarction are CT with contrast, renal angiography, and isotope perfusion scanning with CT scan with contrast being the diagnostic modality of choice; studies would show a wedge-shaped area with unenhanced low peripheral density [10, 13]. On the other hand, ultrasonography has low specificity and sensitivity.

Treatment of ARI depends on the etiology. Anticoagulation, intra-arterial thrombolysis, and surgery are the preferred modalities of management of ARI. In the study of Tsai *et al*., among 18 patients, anticoagulation, local intra-arterial thrombolytic therapies, and intravenous thrombolytic therapies were provided in nine, five and two patients respectively [14]. Korzets *et al*. in their study reported management of three patients with heparin administered intravenously and another with a combination of heparin administered intravenously and renal intra-arterial urokinase infusion; in the latter case, there was no recovery of function of the affected kidney [3]. Anticoagulation can be done with unfractionated heparin, low molecular weight heparin or warfarin after bridging therapy. In our patient, hyperthyroidism induced AF and cardiomyopathy was the probable cause of the thromboembolic phenomenon involving his brain and kidney; he was managed with warfarin and carbimazole.

Irreversible renal damage and hypertension are the well-established complications of renal infarction [15, 16]. However, in our case, there were no signs and symptoms of renal damage and hypertension after 3 months of follow-up.

## Conclusions

ARI is a rare clinical condition with varied nonspecific clinical manifestations. Patients with a cerebrovascular event due to cardiac emboli are to be vigilantly monitored for possible visceral infarctions. A high index of suspicion of renal infarction should be made in a patient with sudden onset flank pain following a cardioembolic stroke in a patient with AF. Hyperthyroidism is a relatively common condition that may result in AF and cardiomyopathy, which increases the risks of thromboembolism.

## Consent

Written informed consent was obtained from the patient for the publication of this case report and any accompanying images. A copy of the written consent is available for review by the Editor-in-Chief of this journal.
